# Identifying and Linking Patients At Risk for MASLD with Advanced Fibrosis to Care in Primary Care

**DOI:** 10.1007/s11606-024-08955-9

**Published:** 2024-07-26

**Authors:** Ted G. Xiao, Lauren Witek, Richa A. Bundy, Adam Moses, Corey S. Obermiller, Andrew D. Schreiner, Ajay Dharod, Mark W. Russo, Sean R. Rudnick

**Affiliations:** 1 Department of Internal Medicine, Wake Forest School of Medicine, Winston-Salem, NC USA; 2 Informatics and Analytics, Department of Internal Medicine, Wake Forest School of Medicine, Winston-Salem, NC USA; 3 Department of Implementation Science, Division of Public Health Science, Wake Forest School of Medicine, Winston-Salem, NC USA; 4 Wake Forest Center for Healthcare Innovation, Wake Forest School of Medicine, Winston-Salem, NC USA; 5 Wake Forest Center for Biomedical Informatics, Wake Forest School of Medicine, Winston-Salem, NC USA; 6 Division of Liver Diseases and Transplant, Atrium Health Carolina Medical Center, Charlotte, NC USA; 7 Section of Gastroenterology and Hepatology, Wake Forest School of Medicine, Winston-Salem, NC USA; 8 Department of Medicine, Medical University of South Carolina, Charleston, SC USA

**Keywords:** MASLD, non-invasive testing, fibrosis-4 score, screening, advanced fibrosis

## Abstract

**Background and Aims:**

Severity of fibrosis is the driver of liver-related outcomes in metabolic dysfunction-associated steatotic liver disease (MASLD), and non-invasive testing such as fibrosis-4 (FIB-4) score is utilized for risk stratification. We aimed to determine if primary care patients at risk for MASLD and advanced fibrosis were evaluated with subsequent testing. A secondary aim was to determine if at-risk patients with normal aminotransferases had advanced fibrosis.

**Methods:**

Primary care patients at increased risk for MASLD with advanced fibrosis (*n* = 91,914) were identified using previously established criteria. Patients with known alternative/concomitant etiology of liver disease or cirrhosis were excluded. The study cohort included patients with calculated FIB-4 score in 2020 (*n* = 52,006), and stratified into low, indeterminate, and high likelihood of advanced fibrosis. Among those at indeterminate/high risk, rates of subsequent testing were measured.

**Results:**

Risk stratification with FIB-4 characterized 77% (*n* = 40,026) as low risk, 17% (*n* = 8847) as indeterminate, and 6% (*n* = 3133) as high risk. Among indeterminate/high-risk patients (*n* = 11,980), 78.7% (*n* = 9433) had aminotransferases within normal limits, 0.95% (*n* = 114) had elastography, and 8.2% (*n* = 984) were referred for subspecialty evaluation.

**Conclusion:**

In this cohort of primary care patients at risk for MASLD with fibrosis, the FIB-4 score identified a substantial proportion of indeterminate/high-risk patients, the majority of which had normal aminotransferase levels. Low rates of subsequent testing were observed. These data suggest that a majority of patients at increased risk for liver-related outcomes remain unrecognized and highlight opportunities to facilitate their identification.

**Supplementary Information:**

The online version contains supplementary material available at 10.1007/s11606-024-08955-9.

## BACKGROUND

The prevalence of non-alcoholic fatty liver disease (NAFLD) in the general population of adults is estimated to be 25–30% and it is generally asymptomatic, hindering early diagnosis.^1^ This number is likely to increase further given the association between NAFLD, type 2 diabetes mellitus (T2DM), and obesity.^2,3^ These associations indeed gave rise to the more descriptive terminology the field has recently shifted to, metabolic dysfunction-associated steatotic liver disease (MASLD) .^4^ Metabolic dysfunction-associated steatohepatitis ^5^ is essentially the subset of MASLD patients with associated steatohepatitis and/or development of fibrosis (formerly NASH). Estimates in the US suggest patients with advanced fibrosis defined as bridging fibrosis (F3) or compensated cirrhosis (F4) will number 7.94 million and account for 29% of MASH cases by 2030.^6^ The fibrosis stage is strongly correlated with liver-related outcomes and death, with advanced fibrosis associated with an exponentially greater risk of liver-related morbidity and mortality than earlier stages of fibrosis.^7,8^ Thus, identifying patients at the highest risk for advanced fibrosis would allow appropriate intervention that may prevent future liver-related complications.^9^

Aminotransferase abnormalities alone do not adequately capture patients with MASLD who have advanced fibrosis.^10^ Non-invasive testing ^11^ includes both indirect (i.e., fibrosis-4 [FIB-4] index) and direct serologic markers (i.e., enhanced liver fibrosis score [ELF]), as well as elastography (i.e., vibration controlled transient elastography [VCTE]) .^12^ Non-invasive testing (NIT) is preferred over liver biopsy for identifying patients at risk for MASLD given that NIT is readily available, and that liver biopsy is an imperfect gold standard that is invasive and impractical at the population level.^11,1,13–15^ The use of indirect NIT allows the screening of patients for which a confirmatory NIT (i.e., subsequent testing) or liver biopsy may be warranted for more definitive evaluation.

Accurately identifying the at-risk population for appropriate testing improves the predictive characteristics of NIT and provides the framework of sequential testing recommended by the American Gastroenterology Association and the European Association for the Study for the Liver.^13,14^ Risk factors for MASLD including T2DM and metabolic syndrome have long been recognized to be associated with an increased risk of MASH and/or advanced fibrosis.^16,17^ Combining FIB-4 score ≥ 1.3 and diabetes as the criterion may minimize the number of indeterminate scores undergoing subsequent VCTE.^13,18^ Some screening algorithms solely utilize NIT for risk stratification to avoid liver biopsy completely.^14^ As most patients at risk for MASLD are seen in primary care clinics, the American Association of Clinical Endocrinology suggests that primary care is the ideal setting for the identification of individuals at risk for advanced fibrosis.^19^

The aims of this study were (1) to determine the prevalence of low, indeterminate, and high risk for MASLD with advanced fibrosis in the primary care setting based upon NIT (FIB-4 score) and the number who underwent subsequent testing for risk stratification (elastography or subspecialty evaluation), and (2) to determine the number of patients with normal aminotransferase levels who had advanced fibrosis based on NIT. Prior work has focused on risk stratification of primary care patients with *known* diagnoses of MASLD. This study included the general primary care population *at risk* for MASLD (with or without preceding diagnosis), thus allowing insight into the detection of advanced fibrosis in previously undiagnosed patients in addition to those with known MASLD.

## METHODS

### Cohort Selection

This retrospective cohort study was conducted at primary care clinics affiliated with an academic tertiary medical center. The target cohort was obtained by applying a set of inclusion and exclusion criteria to identify patients in primary care clinics within the Atrium Health Wake Forest Baptist (AHWFB) primary care network. Patient demographics and clinical administrative data were extracted from the electronic health record (EHR).

The general population in the AHWFB primary care network included all adult patients with a primary care visit during the calendar year of 2020 (*n* = 1,032,812). The primary care network includes approximately 60 outpatient primary care practices comprising over 300 providers. Patients with known alternative and/or concomitant etiology for liver disease and established cirrhosis were excluded based on ICD-10 codes listed in the Supplemental Material (*n* = 244,740). The at-risk population for advanced fibrosis was identified based on criteria outlined by Kanwal and colleagues.^13^ This population (*n* = 91,914) was defined by patients with a (1) diagnosis of T2DM based on ICD-10 codes or (2) at least two of the following metabolic risk factors: obesity (BMI ≥ 30 kg/m^2^), hypertension (based on ICD-10 codes), pre-diabetes (based on ICD-10 codes or HgA1c level between 5.7 and 6.4), triglycerides > 150 mg/dL, reduced HDL (< 40 mg/dL in males, < 50 mg/dL in females). Additionally, patients with ICD-10 codes for NAFLD or NASH (no existing codes for MASLD or MASH at time of the study) and elevated aminotransferase levels (two aminotransferase measurements > upper limit of normal at least 6 months apart) qualified.

**Figure 1:**
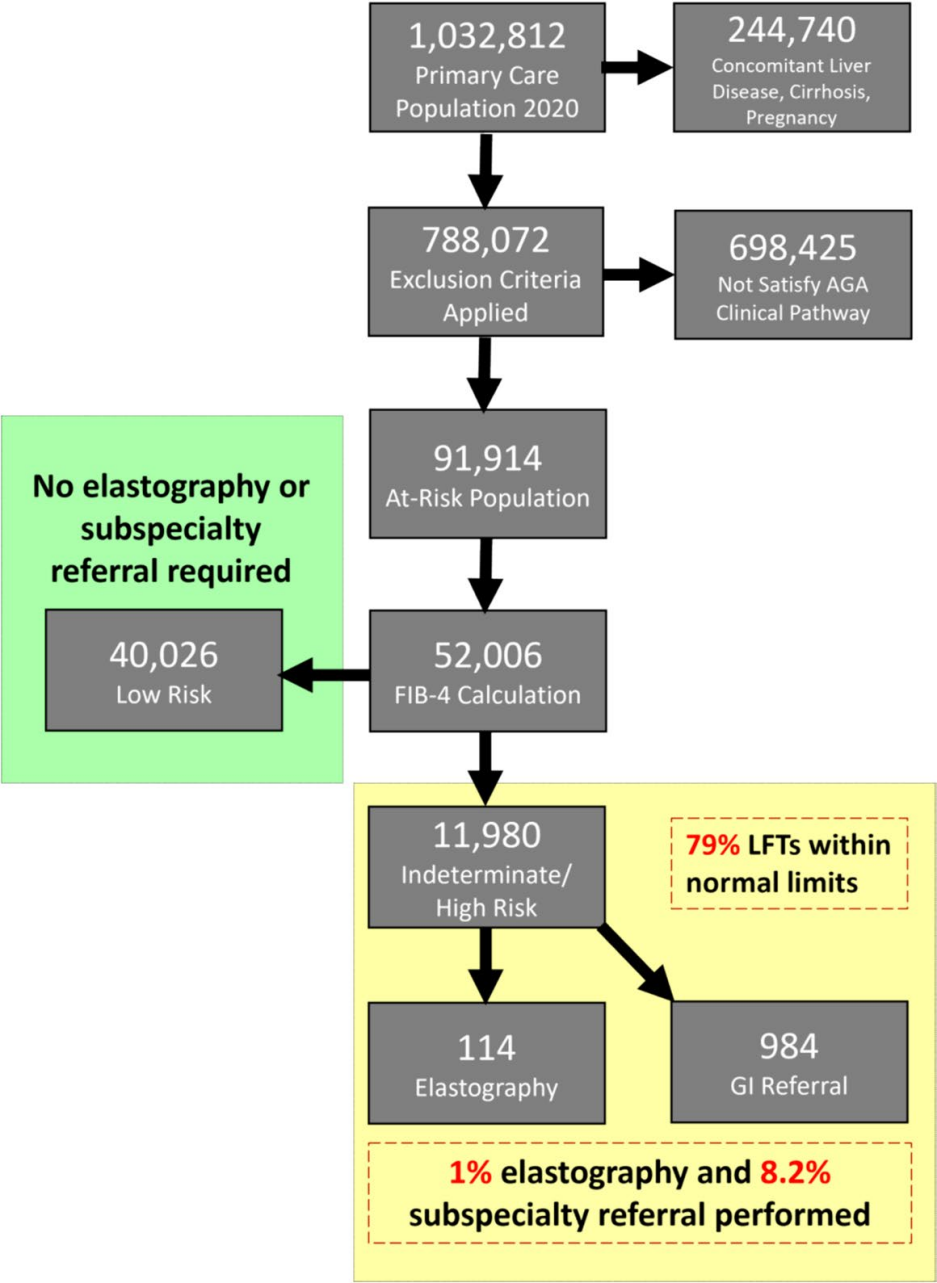
Flow chart of primary care patients at risk for MASLD with advanced fibrosis. Abbreviation: AGA, American Gastroenterological Association; FIB-4, fibrosis-4; GI, gastroenterology.

The study cohort (*n* = 52,006) was identified by filtering for all necessary parameters to calculate the FIB-4 score (age, AST, ALT, platelet count). Inclusion into the study cohort required that laboratory parameters be drawn on the same day within the calendar year 2020. FIB-4 scores were then calculated post hoc. Based upon established cut-offs for the FIB-4 score, the cohort was stratified into low risk (< 1.3 or < 2 for patients ≥ 65 years old), indeterminate risk (1.3–2.67 or 2–2.67 for patients ≥ 65 years old), and high risk (> 2.67) for advanced fibrosis.^20^ Patients with indeterminate or high-risk FIB-4 scores were combined into the “increased risk” group (i.e., recommended to undergo subsequent testing).^13^ Figure 1 is a flow diagram of the cohort selection.

### Outcome Measures

Outcome measures were the proportion of patients at increased risk of advanced fibrosis based on FIB-4 score who received further testing: either elastography or referral for subspecialty evaluation (GI or hepatology), respectively. The subsequent testing had to be performed within 12 months of the date of the calculated FIB-4 score. We also determined the number of patients who had advanced fibrosis based on FIB-4 score with normal aminotransferase levels and characterized those who also had elastography.

We reported frequencies and proportions for all comparisons. Statistical tests included *t*-tests as well as chi-square tests, as appropriate. Data cleaning and statistical analyses were performed using R statistical software, version 4.2.3.

## RESULTS

### Patient Characteristics

A total of 52,006 patients met the inclusion criteria. Demographic information, comorbid medical conditions, mean FIB-4 components, and thrombocytopenia are outlined in Table 1. The average age of the cohort was 62 years and 55.8% were female. The majority of patients identified as white or Caucasian and non-Hispanic/Latino/Spanish (72.8% and 95.8%, respectively). The prevalence of ICD-10 coded NAFLD or NASH in the cohort was 5.3%. The overall prevalence of obesity, pre-diabetes, T2DM, hypertriglyceridemia, and hypertension was 56.3%, 36.1%, 45.7%, 40.0%, and 89.7% respectively. Risk stratification using the FIB-4 score characterized 77.0% (*n* = 40,026) as low risk and 23.0% (*n* = 11,980) as increased risk. Normal aminotransferase levels were observed in 95.4% (*n* = 38,190) of patients at low risk and in 79.3% (*n* = 9506) of patients at increased risk as stratified by the FIB-4 score. There was 23.3% (*n* = 2792) of patients with increased risk based on FIB-4 score with thrombocytopenia compared to 6.0% (*n* = 3097) of the cohort.

**Table 1:**
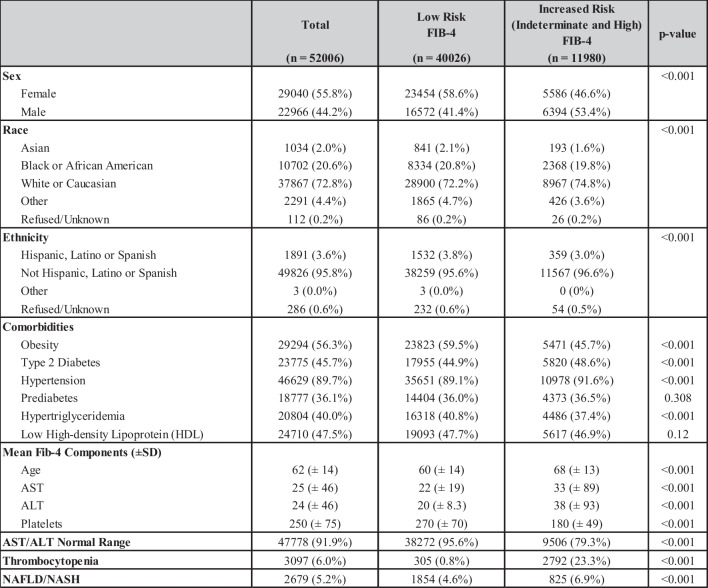
Cohort Characteristics Overall and Stratified into Low and Increased Risk (Those Needed Subsequent Testing) of Advanced Fibrosis Based on FIB-4 Score. Abbreviations: *FIB-4*, fibrosis-4; *NAFLD*, Non-alcoholic fatty liver disease; *NASH*, non-alcoholic steatohepatitis

Thrombocytopenia (defined as platelet count < 150 × 10^9^/L) was present in 6% of the study population (*n* = 3097). Thrombocytopenia was more prevalent among patients with increased risk FIB-4 scores (23.3%) compared to low-risk scores (0.8%).

### Subsequent Testing

Among those with high-risk FIB-4 scores (*n* = 3133), 1.7% (*n* = 53) underwent elastography (VCTE or ultrasound-based) and 8.2% (*n* = 256) were referred for subspecialty evaluation. In patients at indeterminate risk (*n* = 8847), those rates were 0.7% (*n* = 61) and 8.2% (*n* = 728), respectively. When combining indeterminate and high-risk patients into one group (i.e., increased risk), 1.0% (114) had some form of elastography and 8.2% (984) were referred for subspecialty evaluation (Table 2).

**Table 2:**
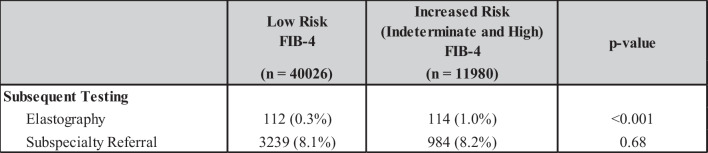
Orders Placed for Subsequent Testing Stratified Based on Low and Increased Risk of Advanced Fibrosis Based on FIB-4 Score. Abbreviations: *FIB-4*, fibrosis-4; *NAFLD*, non-alcoholic fatty liver disease; *NASH*, Non-alcoholic steatohepatitis

### Elastography

Patients with increased risk FIB-4 scores were 3.4 times more likely to have elastography ordered compared to the low-risk FIB-4 group (CI 2.6–4.4; *p* < 0.001). Patients with pre-existing diagnosis of NAFLD or NASH were 16.5 times more likely to have elastography ordered compared to those without the diagnosis (CI 12.8–21.4; *p* < 0.001). Among patients with T2DM, there was no difference in the number of patients undergoing elastography.

Among the 114 patients with FIB-4 score suggesting increased risk of advanced fibrosis who underwent elastography, there were eight invalid studies. Elastography characterized 41.2% (*n* = 47) of them with advanced fibrosis, while 51.8% (*n* = 59) were characterized as no advanced fibrosis. When stratified by FIB-4 score, 54.7% (29/53) of patients with a high FIB-4 score had concordant elastography. In patients with indeterminate FIB-4 scores, 29.5% (18/61) had elastography suggesting advanced fibrosis. There was a small subset of patients with indeterminate/high-risk FIB-4 scores with normal aminotransferase levels who underwent elastography (*n* = 45). Among those, elastography characterized 22.2% (*n* = 10) as having advanced fibrosis.

### Subspecialty Referral

The cohort was stratified based on referral for subspecialty evaluation by FIB-4 score (low vs. increased risk), pre-existing diagnosis of NAFLD or NASH, and presence of T2DM. There was no difference observed in subspecialty referral stratified by FIB-4 score. Patients with a pre-existing diagnosis of NAFLD or NASH were 1.7 times more likely to be referred for subspecialty evaluation (CI 1.6–1.9; *p* ≤ 0.001), while patients with T2DM were 1.1 times more likely to referred (CI 1.03–1.1; *p* = 0.002).

## DISCUSSION

Among a large population of patients evaluated in primary care clinics who are at risk for MASLD and advanced liver fibrosis, approximately one-quarter (23%) have an indeterminate or high-risk FIB-4 score (i.e., increased risk) that warrants subsequent testing for advanced fibrosis. Of those meeting the criteria for further testing, less than 1% underwent elastography, and less than 10% underwent subspecialty evaluation, suggesting a substantial proportion of patients and their providers were unaware of their risk for liver-related morbidity and mortality. These data suggest that patients with MASLD at increased risk for advanced fibrosis are underdiagnosed and are at risk for developing potentially preventable liver-related-complications.

Though risk factors for MASLD are well established, previously published data suggest underdiagnosis of MASLD in the primary care setting.^21,22^ In a large Medicare claims database (> 10 million patients assessed), the reported prevalence of MASLD was 5.7%.^23^ This is supported in the current study as only 5.3% of the cohort at risk for MASLD were carrying an ICD-10 diagnosis of NAFLD or NASH. This is discordant with the previously reported 25% of the general population,^1^ indicating significant under-recognition of the disease. Although not all patients with metabolic risk factors develop MASLD, it is estimated that their risk is 1.3–1.7 times higher than the general population.^24^ In patients with T2DM, the prevalence of MASLD ranges from 30 to 75%.^1^ Despite this strong association, we did not observe significant differences in subsequent testing based on the presence of T2DM, further highlighting under-recognition.

Utilization of a faulty diagnostic heuristic could be a potential reason for the under-recognition of MASLD. While using the FIB-4 scores to identify at-risk patients is well-established, previous studies suggest that providers may instead rely upon liver enzymes as a screening heuristic for MASLD.^22^ Elevated aminotransferase levels should alarm providers to investigate hepatic injury, but normal aminotransferase levels do not necessarily reflect the absence of liver disease. In fact, aminotransferase levels can be normal in patients with MASLD/MASH and advanced fibrosis and cirrhosis on biopsy.^10,25,26^ In the present study, just under one-quarter (22%) of patients with normal aminotransferase levels had advanced fibrosis/cirrhosis based on elastography. Alarmingly, 64.2% of patients in our study with high FIB-4 scores had aminotransferase levels within the normal range (as defined by their local lab). Thus, it is unlikely those patients would have “raised suspicion” in their PCPs of underlying liver disease, highlighting a major shortcoming of relying on aminotransferase levels alone.

Only recently have the identified risk factors and the sequential use of NIT in screening patients at risk been codified into recommendations by US professional societies.^13^ However, specific risk factors for MASLD (i.e., T2DM, metabolic syndrome) have long been recognized to be associated with an increased risk of MASH and/or advanced fibrosis, and the diagnostic utility of the FIB-4 score for identifying at-risk patients was well-established prior to the publication of the 2021 ACG Clinical Pathway.^17,27^ The main objective in primary care clinics is to identify the large proportion of MASLD patients at low risk for advanced fibrosis, as only a minority (3–5%) of patients with MASLD will progress to cirrhosis.^28,29^ In this setting, the excellent negative predictive value (~ 90%) of the widely available/cost-effective FIB-4 score can exclude advanced fibrosis and identify low-risk patients who do not require additional testing.^13,30^ Though these patients are at decreased risk of liver-related morbidity/mortality compared to those with higher degrees of fibrosis, they remain at risk for death from cardiovascular disease and non-hepatic malignancy.^1^ In this population, management of metabolic syndrome components may both decrease the risk of fibrosis progression and improve non-liver-related morbidity/mortality.

The FIB-4 score identifies a substantial proportion of patients in the indeterminate or high-risk category (i.e., increased risk) needing further evaluation. In the US, it is estimated that 20.3 million patients would be categorized as indeterminate or high-risk based on the FIB-4 score.^18^ Due to the low positive predictive value of FIB-4 score for advanced fibrosis, especially within the indeterminate population, subsequent assessment strategies such as elastography or serum tests are recommended to evaluate for liver fibrosis.^13,14^ In those patients undergoing further assessment (i.e., elastography), it is suggested those patients who remain at indeterminate or high risk and/or have discordant findings undergo additional testing (i.e., subspecialty referral and/or liver biopsy). The objective in this population is to identify patients with advanced fibrosis/cirrhosis so targeted and evidence-based interventions that improve morbidity and mortality can be appropriately initiated (i.e., variceal and hepatocellular carcinoma screening). Thus, identifying patients with advanced liver fibrosis has important clinical implications. Prior population-based data suggest approximately 4% of patients with indeterminate FIB-4 scores will have elastography suggesting advanced fibrosis, thus re-classifying these patients as high-risk.^18^ If our entire indeterminate risk population (*n* = 8847) were screened with elastography, there would be an additional 354 patients diagnosed with advanced fibrosis. However, in our study, 0.7% (*n* = 61) of the entire indeterminate risk population was screened and only 18 patients were diagnosed with advanced fibrosis, leaving a majority of patients with advanced fibrosis undiagnosed.

With the rising prevalence of MASLD, we can expect further strain on limited healthcare resources and exacerbation of the already substantial economic burden.^31,32^ Given the large number of patients with MASLD, a common clinical question raised by PCPs is which patients to refer for subspecialty evaluation. A 2018 prospective longitudinal cohort study from the UK implemented a two-step screening pathway in the primary care setting utilizing FIB-4 score as the initial screening test. Prior to introduction of the pathway, 66% of patients referred for subspecialty evaluation had a baseline FIB-4 score suggesting low risk of advanced fibrosis (i.e., referral could have been avoided).^33^ Following implementation, they observed an 80% decrease in unnecessary referrals. Furthermore, the pathway increased the detection of advanced fibrosis and cirrhosis five-fold and three-fold, respectively. These real-world data demonstrate that implementing screening pathways will likely optimize use of the limited available resources. This group also demonstrated the cost efficiency of such screening programs.^34^

Limitations to our study must be acknowledged. The study period preceded the publication of the American Gastroenterology Association (AGA) Clinical Care Pathway in 2021, which outlines a strategy for screening at-risk patients.^13^ Available data did not allow determination of the frequency with which PCPs calculated FIB-4 scores in clinical practice during the study period (calculated post hoc). This raises two troublesome but equally important possibilities. First, our data may reflect the previously reported low utilization of FIB-4 scores in clinical practice among PCPs.^35^ Furthermore, the low number of patients with elevated liver chemistries likely resulted in the under-recognition of at-risk patients. Alternatively, if one were to assume FIB-4 scores were calculated with regularity during the study period, the data would suggest inadequate resources to appropriately link identified at-risk patients to subsequent testing. Additionally, the study cohort was followed in calendar year 2020, during the height of the COVID-19 pandemic which may have influenced practice patterns. Reassuringly, similar data collected from 2012 to 2021 (encompassing 8 years of data collection not affected by the COVID-19 pandemic) demonstrated that 10.3% of patients underwent confirmatory testing, similar to the observed rates in the present study.^36^ Another limitation of the study is that the specific indication for subspecialty referral was not known in all patients. Thus, it is likely that a proportion of referrals observed were in fact for diagnoses other than MASLD (i.e., unrelated to liver disease). This would suggest that MASLD and advanced fibrosis as a reason for referral was even lower than the ~ 8% observed. We acknowledge that the generalizability of the results may be limited as this study was performed within a single healthcare system; however, our study included over 300 providers at 60 primary care practices. Additionally, the race/ethnic composition of our population may not be representative of other geographic locations and/or healthcare systems.

Our study has several strengths. In attempts to overcome the inherent limitation of using ICD-10 codes, our study created a computational algorithm in the EHR (electronic phenotyping) for MASLD with risk for advanced fibrosis. We included utilizing source data from the EHR when possible (i.e., BMI measurements, measured hemoglobin A1c levels, cholesterol levels, etc.). This likely improved accuracy in the identification of the at-risk population. Compared to similar work, our study included a larger study population because the analysis was not limited to patients previously diagnosed with MASLD, but rather included the entire primary care population at risk.^36^ Thus, we were able to identify previously undiagnosed patients with MASLD at risk for advanced fibrosis in addition to those already diagnosed with MASLD.

The present study suggests that patients with MASLD at risk for liver-related outcomes remain under-identified, potentially due to dependence on a historical heuristic (elevated aminotransferase levels). The electronic phenotyping of MASLD and advanced fibrosis within this study also highlights an opportunity for screening tools for patients at risk for MASLD. The computational algorithm for identification of patients with MASLD and MASH at risk for advanced fibrosis provides a foundation for further investigation of EHR-based implementation studies. Acknowledging the significant responsibilities and time constraints of PCPs, along with patient-level factors, we hypothesize developing EHR-based clinical decision support (CDS) tools facilitating screening and appropriate subsequent testing for patients will be embraced by patients, providers, and health systems. Such processes may also allow for optimizing the efficient use of resources to ensure those with MASLD at the highest risk of advanced fibrosis are identified and appropriately managed to prevent liver-related morbidity and mortality.

## Supplementary Information

Below is the link to the electronic supplementary material.

Supplementary file1 (DOCX 22 KB)

## Data Availability

De-identified source data will be maintained internally and can be accessed based upon reader questions or concerns.

## Abbreviations

NAFLD: Non-alcoholic fatty liver disease
NASH: Non-alcoholic steatohepatitis
MASLD: Metabolic dysfunction-associated steatotic liver disease
FIB-4: Fibrosis-4
T2DM: Type 2 diabetes mellitus
ELF: Enhanced liver fibrosis
VCTE: Vibration controlled transient elastography
NPV: Negative predictive value
NIT: Non-invasive testing
EHR: Electronic health record
CDS: Clinical decision support

## References

[1] **Rinella ME, Neuschwander-Tetri BA, Siddiqui MS, et al.** AASLD Practice Guidance on the clinical assessment and management of nonalcoholic fatty liver disease. Hepatology (Baltimore, Md). 2023;77(5):1797-1835. 10.1097/hep.0000000000000323. 36727674 10.1097/HEP.0000000000000323 PMC10735173

[2] **Blais P, Husain N, Kramer JR, Kowalkowski M, El-Serag H, Kanwal F.** Nonalcoholic fatty liver disease is underrecognized in the primary care setting. Am J Gastroenterol. 2015;110(1):10-4. 10.1038/ajg.2014.134. 24890441 10.1038/ajg.2014.134

[3] **Hales CM, Carroll MD, Fryar CD, Ogden CL.** Prevalence of Obesity Among Adults and Youth: United States, 2015-2016. NCHS Data Brief. 2017;(288):1-8. 29155689

[4] **Rinella ME, Lazarus JV, Ratziu V, et al.** A multi-society Delphi consensus statement on new fatty liver disease nomenclature. Hepatology (Baltimore, Md). 2023; 10.1097/hep.0000000000000520. 10.1097/HEP.0000000000000696 37983810

[5] **Yamashita F, Hashida M.** Pharmacokinetic considerations for targeted drug delivery. Adv Drug Deliv Rev. 2013;65(1):139-47. 10.1016/j.addr.2012.11.006. 23280371 10.1016/j.addr.2012.11.006

[6] **Estes C, Razavi H, Loomba R, Younossi Z, Sanyal AJ.** Modeling the epidemic of nonalcoholic fatty liver disease demonstrates an exponential increase in burden of disease. Hepatology (Baltimore, Md). 2018;67(1):123-133. 10.1002/hep.29466. 28802062 10.1002/hep.29466 PMC5767767

[7] **Taylor RS, Taylor RJ, Bayliss S, et al.** Association Between Fibrosis Stage and Outcomes of Patients With Nonalcoholic Fatty Liver Disease: A Systematic Review and Meta-Analysis. Gastroenterology. 2020;158(6):1611-1625.e12. 10.1053/j.gastro.2020.01.043. 32027911 10.1053/j.gastro.2020.01.043

[8] **Sanyal AJ, Van Natta ML, Clark J, et al.** Prospective Study of Outcomes in Adults with Nonalcoholic Fatty Liver Disease. N Engl J Med. 2021;385(17):1559-1569. 10.1056/NEJMoa2029349. 34670043 10.1056/NEJMoa2029349 PMC8881985

[9] **Jarvis H, Craig D, Barker R, et al.** Metabolic risk factors and incident advanced liver disease in non-alcoholic fatty liver disease (NAFLD): A systematic review and meta-analysis of population-based observational studies. PLoS Med. 2020;17(4):e1003100. 10.1371/journal.pmed.1003100. 32353039 10.1371/journal.pmed.1003100 PMC7192386

[10] **Mofrad P, Contos MJ, Haque M, et al.** Clinical and histologic spectrum of nonalcoholic fatty liver disease associated with normal ALT values. Hepatology (Baltimore, Md). 2003;37(6):1286-92. 10.1053/jhep.2003.50229. 12774006 10.1053/jhep.2003.50229

[11] **Kanwal F, Kramer J, Asch SM, et al.** An explicit quality indicator set for measurement of quality of care in patients with cirrhosis. Clin Gastroenterol Hepatol. 2010;8(8):709-17. 10.1016/j.cgh.2010.03.028. 20385251 10.1016/j.cgh.2010.03.028

[12] **Tapper EB, Lok AS.** Use of Liver Imaging and Biopsy in Clinical Practice. N Engl J Med. 2017;377(8):756-768. 10.1056/NEJMra1610570. 28834467 10.1056/NEJMra1610570

[13] **Kanwal F, Shubrook JH, Adams LA, et al.** Clinical Care Pathway for the Risk Stratification and Management of Patients With Nonalcoholic Fatty Liver Disease. Gastroenterology. 2021;161(5):1657-1669. 10.1053/j.gastro.2021.07.049. 34602251 10.1053/j.gastro.2021.07.049 PMC8819923

[14] EASL Clinical Practice Guidelines on non-invasive tests for evaluation of liver disease severity and prognosis - 2021 update. Hepatol. 2021;75(3):659–689. 10.1016/j.jhep.2021.05.025. 10.1016/j.jhep.2021.05.025 34166721

[15] **Mehta SH, Lau B, Afdhal NH, Thomas DL.** Exceeding the limits of liver histology markers. J Hepatol. 2009;50(1):36-41. 10.1016/j.jhep.2008.07.039. 19012989 10.1016/j.jhep.2008.07.039 PMC2637134

[16] **Angulo P, Hui JM, Marchesini G, et al.** The NAFLD fibrosis score: a noninvasive system that identifies liver fibrosis in patients with NAFLD. Hepatology (Baltimore, Md). 2007;45(4):846-54. 10.1002/hep.21496. 17393509 10.1002/hep.21496

[17] EASL-EASD-EASO Clinical Practice Guidelines for the management of non-alcoholic fatty liver disease. J Hepatol. 2016;64(6):1388–402. 10.1016/j.jhep.2015.11.004. 10.1016/j.jhep.2015.11.004 27062661

[18] **Udompap P, Therneau TM, Canning RE, Benson JT, Allen AM.** Performance of American Gastroenterological Association Clinical Care Pathway for the risk stratification of patients with nonalcoholic fatty liver disease in the US population. Hepatology (Baltimore, Md). 2023;77(3):931-941. 10.1002/hep.32739. 35989502 10.1002/hep.32739

[19] **Cusi K, Isaacs S, Barb D, et al.** American Association of Clinical Endocrinology Clinical Practice Guideline for the Diagnosis and Management of Nonalcoholic Fatty Liver Disease in Primary Care and Endocrinology Clinical Settings: Co-Sponsored by the American Association for the Study of Liver Diseases (AASLD). Endocr Pract. 2022;28(5):528-562. 10.1016/j.eprac.2022.03.010. 35569886 10.1016/j.eprac.2022.03.010

[20] **McPherson S, Hardy T, Dufour JF, et al.** Age as a Confounding Factor for the Accurate Non-Invasive Diagnosis of Advanced NAFLD Fibrosis. Am J Gastroenterol. 2017;112(5):740-751. 10.1038/ajg.2016.453. 27725647 10.1038/ajg.2016.453 PMC5418560

[21] **Nielsen EM, Anderson KP, Marsden J, Zhang J, Schreiner AD.** Nonalcoholic Fatty Liver Disease Underdiagnosis in Primary Care: What Are We Missing? J Gen Intern Med. 2022;37(10):2587-2590. 10.1007/s11606-021-07197-3. 34816326 10.1007/s11606-021-07197-3 PMC9360350

[22] **Saeed N, Glass LM, Habbal H, et al.** Primary care and referring physician perspectives on non-alcoholic fatty liver disease management: a nationwide survey. Therap Adv Gastroenterol. 2021;14:17562848211042200. 10.1177/17562848211042200. 34567270 10.1177/17562848211042200 PMC8460969

[23] **Loomba R, Wong R, Fraysse J, et al.** Nonalcoholic fatty liver disease progression rates to cirrhosis and progression of cirrhosis to decompensation and mortality: a real world analysis of Medicare data. Aliment Pharmacol Ther. 2020;51(11):1149-1159. 10.1111/apt.15679. 32372515 10.1111/apt.15679

[24] **Payne JY, Alkhouri N, Le P, et al.** Prevalence of at-risk NASH and its association with metabolic syndrome in US adults with NAFLD, 2017-2018. Hepatol Commun. 2023;7(1):e0019. 10.1097/hc9.0000000000000019. 36633494 10.1097/HC9.0000000000000019 PMC9833447

[25] **Kasarala G, Tillmann HL.** Standard liver tests. Clin Liver Dis (Hoboken). 2016;8(1):13-18. 10.1002/cld.562. 31041056 10.1002/cld.562 PMC6490189

[26] **Verma S, Jensen D, Hart J, Mohanty SR.** Predictive value of ALT levels for non-alcoholic steatohepatitis (NASH) and advanced fibrosis in non-alcoholic fatty liver disease (NAFLD). Liver Int. 2013;33(9):1398-405. 10.1111/liv.12226. 23763360 10.1111/liv.12226

[27] **Guha IN, Parkes J, Roderick P, et al.** Noninvasive markers of fibrosis in nonalcoholic fatty liver disease: Validating the European Liver Fibrosis Panel and exploring simple markers. Hepatology (Baltimore, Md). 2008;47(2):455-60. 10.1002/hep.21984. 18038452 10.1002/hep.21984

[28] **Vernon G, Baranova A, Younossi ZM.** Systematic review: the epidemiology and natural history of non-alcoholic fatty liver disease and non-alcoholic steatohepatitis in adults. Aliment Pharmacol Ther. 2011;34(3):274-85. 10.1111/j.1365-2036.2011.04724.x. 21623852 10.1111/j.1365-2036.2011.04724.x

[29] **Allen AM, Therneau TM, Ahmed OT, et al.** Clinical course of non-alcoholic fatty liver disease and the implications for clinical trial design. J Hepatol. 2022;77(5):1237-1245. 10.1016/j.jhep.2022.07.004. 35843374 10.1016/j.jhep.2022.07.004 PMC9974107

[30] **Shah AG, Lydecker A, Murray K, Tetri BN, Contos MJ, Sanyal AJ.** Comparison of noninvasive markers of fibrosis in patients with nonalcoholic fatty liver disease. Clin Gastroenterol Hepatol. 2009;7(10):1104-12. 10.1016/j.cgh.2009.05.033. 19523535 10.1016/j.cgh.2009.05.033 PMC3079239

[31] **Younossi ZM, Blissett D, Blissett R, et al.** The economic and clinical burden of nonalcoholic fatty liver disease in the United States and Europe. Hepatology (Baltimore, Md). 2016;64(5):1577-1586. 10.1002/hep.28785. 27543837 10.1002/hep.28785

[32] **Younossi ZM, Tampi RP, Racila A, et al.** Economic and Clinical Burden of Nonalcoholic Steatohepatitis in Patients With Type 2 Diabetes in the U.S. Diabetes Care. 2020;43(2):283–289. 10.2337/dc19-1113. 10.2337/dc19-1113 31658974

[33] **Srivastava A, Gailer R, Tanwar S, et al.** Prospective evaluation of a primary care referral pathway for patients with non-alcoholic fatty liver disease. J Hepatol. 2019;71(2):371-378. 10.1016/j.jhep.2019.03.033. 30965069 10.1016/j.jhep.2019.03.033

[34] **Srivastava A, Jong S, Gola A, et al.** Cost-comparison analysis of FIB-4, ELF and fibroscan in community pathways for non-alcoholic fatty liver disease. BMC Gastroenterol. 2019;19(1):122. 10.1186/s12876-019-1039-4. 10.1186/s12876-019-1039-4 PMC6624894 31296161

[35] **Islam KB, Brandman D, Chu JN, Goldman ML, Fox RK.** Primary Care Providers and Nonalcoholic Fatty Liver Disease: A Needs Assessment Survey. Dig Dis Sci. 2023;68(2):434-438. 10.1007/s10620-022-07706-2. 36178567 10.1007/s10620-022-07706-2 PMC9905186

[36] **Moore JA, Wheless WH, Zhang J, et al.** Gaps in Confirmatory Fibrosis Risk Assessment in Primary Care Patients with Nonalcoholic Fatty Liver Disease. Dig Dis Sci. 2023; 10.1007/s10620-023-07959-5. 10.1007/s10620-023-07959-5 PMC10659111 37193930

