# Supplementary material for: Identifying and Linking Patients At Risk for MASLD with Advanced Fibrosis to Care in Primary Care
Source: J Gen Intern Med. 2024 Jul 26;40(3):629–36. doi: 10.1007/s11606-024-08955-9 (PMC11861828; doi:10.1007/s11606-024-08955-9)
Supplement: Supplementary file 1 — Supplementary file1 (DOCX 22 KB) [file 11606_2024_8955_MOESM1_ESM.docx]

**Supplemental Material**

Supplemental Table 1: ICD-10 codes for concomitant liver disease etiologies that were excluded.

Supplemental Table 2: Elastography orders stratified based on low and increased risk of advanced fibrosis based on FIB-4 score and based on comorbidities. Abbreviations: FIB-4 = fibrosis-4, NAFLD = nonalcoholic fatty liver disease, NASH = nonalcoholic steatohepatitis

Supplemental Table 3: Subspecialty referral orders stratified based on low and increased risk of advanced fibrosis based on FIB-4 score and based on comorbidities. Abbreviations: FIB-4 = fibrosis-4, NAFLD = nonalcoholic fatty liver disease, NASH = nonalcoholic steatohepatitis
